# UV-Vis Spectrophotometrical and Analytical Methodology for the Determination of Singlet Oxygen in New Antibacterials Drugs

**Published:** 2007-11-11

**Authors:** Tamara Zoltan, Franklin Vargas, Carla Izzo

**Affiliations:** Laboratorio de Fotoquímica, Centro de Química, Instituto Venezolano de Investigaciones Científicas, Apartado 21827, Caracas 1020-A, Venezuela.

**Keywords:** histidine assay, singlet oxygen, anti-bacterial, photochemical activity, quinolone, reactive oxygen species

## Abstract

We have determined and quantified spectrophotometrically the capacity of producing reactive oxygen species (ROS) as ^1^O_2_ during the photolysis with UV-A light of 5 new synthesized naphthyl ester derivates of well-known quinolone antibacterials (nalidixic acid (**1**), cinoxacin (**2**), norfloxacin (**3**), ciprofloxacin (**4**) and enoxacin (**5**)). The ability of the naphthyl ester derivatives (**6**–**10**) to generate singlet oxygen were detecting and for the first time quantified by the histidine assay, a sensitive, fast and inexpensive method. The following tendency of generation of singlet oxygen was observed: compounds **7** > **10** > **6** > **8** > **9** >> parent drugs **1**–**5**.

## Introduction

**Figure 1:**
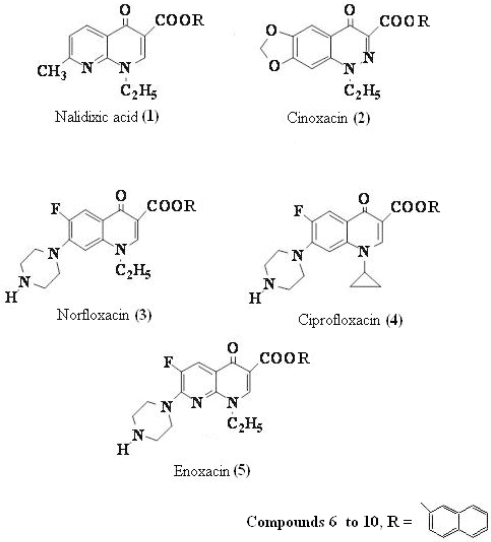
Structure of some of the first and second generation antibacterial quinolones (**1**–**5**) and the new synthesized naphthyl ester derivates **6** to **10**.

Quinolones are antibacterial agents, whose pharmacological action involves the inhibition of an enzyme (bacterial topoisomerase DNA gyrase) that controls the shape of DNA (Sauvaigo et al 2001). A major side effect of these drugs is skin photosensitization (Vargas et al. 2003; Vargas and Rivas, 1997; Tokura 1998; Naldi et al 1999; Arata et al 1998). This has stimulated the photophysical, photochemical and photobiological studies on a large number of quinolones, such as nalidixic acid (**1**), cinoxacin (**2**), norfloxacin (**3**), ciprofloxacin (**4**) and enoxacin (**5**) and their new naphthyl ester derivatives (**6**–**10**) respectively (Fig. 1).

Quinolones undergo a variety of photochemical processes such as decarboxylation, defluorination, oxidation of an amino substituent at C-7, generation of singlet oxygen and production of superoxide (Vargas et al 2006 and 1991; Vargas 1997; Fasani et al 1998; Martinez et al 1997). These oxygenated species (^1^O_2_ and ^•−^O_2_) can alter the oxidant - antioxidant balance of the biological system through different processes broadly studied as the lipid peroxidation, hemolysis of erythrocytes and damages to neutrophils and DNA (photo-genotoxicity). Singlet oxygen can be generated inside cells by photosensitization and can react efficiently with DNA. An understanding of the genotoxic potential of singlet oxygen requires insight into the following parameters: generation of singlet oxygen and bioavailability to DNA; the reactivity of singlet oxygen with DNA and the nature of the modifications induced; mutagenecity and repair of the DNA modifications induced; and secondary products generated from singlet oxygen that could account for any indirect mutagenecity.

On the other hand, recent joint efforts of physicists, chemists, and physicians resulted in a significant progress in photodynamic therapy (PDT) of malignant tumors and some non-oncological diseases (Privalov et al 2002; Dougherty 2002). This therapy involves the administration of a photosensitizing agent followed by tissue exposure to a sufficiently powerful laser irradiation in the visible range. When the tumor with accumulated photosensitizer is illuminated by light of the appropriate wavelength, photochemical reactions occur. Most probably, a light induced excitation of the photosensitizer molecules produces a series of molecular energy transfers to ground state oxygen. This last process leads to a generation of singlet molecular oxygen (^1^O_2_), highly reactive and cytotoxic species, resulting in cell death.

The singlet oxygen quantum yield (φs) is a key property of a photosensitizing agent. This quantity is defined as the number of molecules of ^1^O_2_ generated for each photon absorbed by a photosensitizer. Quantum efficiency is an equivalent term. The production of ^1^O_2_ by photosensitization involves four steps: (A) Absorption of light by the photosensitizer; (B) Formation of the photosensitizer triplet state; the quantum yield of this process is the ISC efficiency or triplet yield (φt); (C) Trapping of the triplet state by molecular oxygen within its lifetime; the fraction of trapped triplet states in a given system is designated by Ft; (D) Energy transfer (Et) from the triplet state to molecular oxygen; the probability of this energy transfer is Et; the experimental value of Et is usually unity for those agents in which the fluorescence is not quenched by oxygen. Overall, φs = φt · Ft · Et. Virtually all measurements of φs are scaled to a reference substance. Frequently employed standard values of φs in aqueous media are 0.79 for rose Bengal, 0.52 for methylene blue, and 1.00 for fullerene C60. The published values of φs show considerable variations with the solvent, reaction conditions, and the measurement technique (Redmond and Gamlin 1999).

The photophysical and photochemical behavior of photosensitizing drugs under aerobic conditions is particularly relevant to understand the *in vivo* photobiological effects. In this context, it is somewhat surprising that ofloxacin and rufloxacin, in spite of their remarkable structural similarity, appear to follow diverging photoreactivity patterns in the presence of oxygen. Thus for example, rufloxacin has a singlet oxygen quantum yield two times higher than that observed for ofloxacin (Sortino et al 1999; Navaratnam and Claridge 2000).

Expensive methodology or technique used to measure the singlet oxygen yield are given from direct detection of the luminescence produced on relaxation of singlet oxygen (time-resolved or steady-state), calorimetric techniques (photoacoustic calorimetry) and time resolved thermal lensing. It is necessary to perform the appropriate screening for phototoxicity *in vitro* before introducing drugs and chemicals into clinical therapy. The use of human erythrocytes, lymphocytes and/or neutrophils as cellular systems in our investigation, combined with other *in vitro* tests employing linoleic acid for lipid photoperoxidation and histidine assay, a sensitive spectrophotometric method to the determination and now for the first time quantified of singlet oxygen, confirmed an important methodology for the study of the phototoxicity of drugs.

The aim of the present study was to establish the histidine assay as a sensitive, fast and inexpensive method to the quantification of singlet oxygen generation by quinolone antibacterials, with special emphasis on the new synthesized naphthyl ester derivative **6** to **10**. On the other hand, the synthesis of the quinolone homologous naphthyl ester derived was carried out with the intention of increasing their photostability and their fluorescence quantum yield (whose fact was achieved) and in this way to give to the quinolones better properties of energy transfer when they are subjected to irradiation. This would generate in these compounds, apart from their antibacterials properties, a new behavior and utility like photosensitizer in bacterial media.

## Materials and Methods

### Chemicals

Nalidixic acid (**1**), cinoxacin (**2**), norfloxacin (**3**) were purchased from Sigma-Aldrich (St. Louis, MO, USA), while ciprofloxacin (**4**) and enoxacin (**5**) were purchased from Fluka-Riedel-deHaën (Buchs, SG, Schweiz). Histidine and p-nitrosodimethylaniline were purchased from Aldrich (Milwaukee, USA). All analytical or HPLC grade solvents were obtained from Merck (Darmstadt, Germany). Their purity was 99.2% as determined by ^1^H NMR-spectroscopy (Bruker Aspect 3000, 300 MHz). The spectrophotometrical experiments were recorded with an UV-Vis-Lambda650 spectrophotometer and a Luminescence Spectrometer LS45 Perkin Elmer. The structures of the isolated products were elucidated by ^1^H NMR and ^13^C NMR (Brucker Aspect 3000, 300 and 100 MHZ respectively), I.R. (Nicolet DX V 5.07).

### Synthesis of naphthyl ester quinolone derivatives

The synthesis for obtaining the ester compounds were developed with some modifications, taking as example the methodology of the patents Bayer Aktiengesellschaft and Italian Pulitzer S.p.A. (Bayer Aktiengesellschaft Patent 557550, 1987; Pulitzer Italiana S.p.A. Patent 537810, 1984). The esterification of quinolones **1** to **5** with β-naphthol was carried out by making pass a flow of dry and gassy HCL through an equimolar dispersion of the corresponding quinolone and β-naphthol (3.0 × 10^−3^ mol) in CH_2_Cl_2_ at the reflux temperature during 1 hour. The solid filtrate and washed with dichloromethane were dissolved in cold water and taken to pH 8.9–9.2 (accurately), where a solid is precipitate in cold overnight.

### Irradiation

All processes of irradiation were carried out using a illuminator Cole Palmer 41720-series keeping a distance of 10 cm between the lamp surface and the solution, varying the time periods of exposure at 25 °C under continuous shaking, with a emission maximum in UVA-Vis 320–400 nm (3.3 mW/cm^2^, 45.575 Lux/seg) (radiation dose 4.5 J/cm^2^) as measured with a model of UVX Digital Radiometer after 1 h continued illumination.

### Singlet oxygen generation and quantification

Photosensitized degradation of histidine was measured in the presence of 0.25, 0.50, 1.0, and 1.5 × 10^−5^ M solution of compounds **1** to **10** (in etanol/H_2_O 1:10). These solutions were mixed with an equal quantity of L-histidine solution at 0.60 to 0.74 mM in phosphate buffer 0.01 M, pH 7.4. Samples of these mixtures were irradiated with an illuminator Cole Palmer 41720-series keeping a distance of 10 cm between the lamp surface and the solution at 25 °C, with a emission maximum in UV-A-Vis 320–400 nm (3.3 mW/cm^2^, 45.575 Lux/seg) at time intervals from 45 to 60 min, with the respective controls being protected from light. The concentration of histidine was determined by a colorimetric reaction. The optic density was read on a spectrophotometer at 440 nm against a blank reagent (L-histidine/p-nitrosodimethylaniline/quinolone derivatives without irradiation) by bleaching of p-nitrosodimethylaniline (Lovell and Sanders 1990; Kraljic and El Mohsni 1978). Rose Bengal, a well known ^1^O_2_ sensitizer, was used as a standard for comparison with the compounds **1** to **10** for ^1^O_2_ formation, under identical conditions of photolysis. The quantum yield of singlet oxygen generation for Rose Bengal is φ(^1^O_2_) = 0.76 (Redmond and Gamlin 1999). This value can be used as a standard to determine a relative quantum yield of the new compounds.

### Statistical treatment of results

At least three independent experiments were performed except where indicated otherwise. The results of the quantification are expressed as a mean ± S.D. Standard deviation (S.D.) is obtained from 3–4 observations. The level of significance accepted was p ≤ 0.05. Statistical analyses were performed using *t*-test.

## Results and Discussion

The synthesis of the compounds **6** to **10** were carried out taking as example the procedures of the patented works of Bayer Aktiengesselschaft and Italian Pulitzer S.p.A. with some modifications. Next some physical corrected and spectroscopics data are presented.

The corresponding naphthyl ester (**6**); 1-ethyl-1,4-dihydro-7-methyl-4-oxo-1,8-naphthyridine-3-naphthyl ester (C_22_H_18_N_2_O_3_, mol wt 358.39), yield: 0.520 g of yellow needles (50.62%), m.p. 127–130 °C. I.R. (KBr): 3205, 1936, 1697, 1616, 1352, 1255, 1220, 744 cm^−1^.

^1^H-NMR (300 MHz, CDCl_3_): δ = ppm 8.86 (d, 1H, J_6–5_ = 7.30, H-6), 8.65 (m, 1H, H-19), 7.73 (s, 1H, H-10), 7.68 (d, 1H, J_18–19_ = 9.00, H-18), 7.60 (m, 1H, H-26), 7.37 (m, 1H, H-22), 7.24 (m, 1H, H-24), 7.17 (m, 1H, H-23), 7.11 (m, 1H, H-25), 6.80 (d, 1H, J_5–6_ = 7.30, H-5), 4.52 (q, 2H, J_11–12_ = 6.80, H-11), 2.70 (s, 3H, H-13), 1.40 (t, 3H, J_12–11_ = 6.80, H-12).

^13^C-NMR (300 MHz, CDCl_3_): δ = 178.66 (CO-8), 167.28 (C-2), 164.77 (CO-15), 153.00 (C-17), 148.58 (C-4), 148.20 (C-10), 136.05 (C-21), 134.06 (C-6), 129.65 (C-19), 128.74 (C-23), 127.64 (C-26), 126.00 (C-25), 126.31 (C-24), 123.34 (C-5), 122.29 (C-18), 111-22), 109.46 (C-9), 47.44 (C-11), 25.29 (C-13), 15.16 (C-12).

The corresponding naphthyl ester (**7**); 1-ethyl-1,4-dihydro-4-oxo-[1,3]dioxolo[4,5-g]cinnoline-3-naphthyl ester (C_22_H_16_N_2_O_5_, mol wt 388.37), yield: 0.490 g of yellow needles (42%), m.p. 143–145 °C. I.R. (KBr): 3282, 1700, 1631, 1469, 1385, 1276, 1242, 743 cm^−1^.

**Figure:**
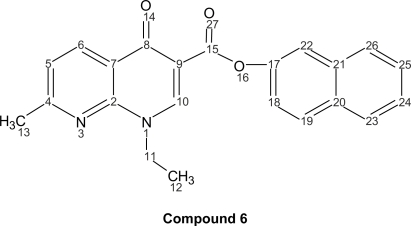


^1^H-NMR (300 MHz, CDCl_3_): δ = ppm 7.75 (d, 1H, H-20), 7.67 (d, 1H, H-19), 7.63 (s, 1H, H-23), 7.59 (m, 1H, H-27), 7.37 (m, 1H, H-25), 7.29 (m, 1H, H-24), 7.10 (m, 1H, H-26), 6.50 (s, 1H, H-13), 6.21 (s, 1H, H-6), 5.66 (s, 2H, H-2), 4.56 (q, 2H, J_14–15_ = 6.88, H-14), 1.50 (t, 3H, J_15–14_ = 6.88, H-15). ^13^C-NMR (300 MHz, CDCl_3_): δ = 169.78 (C-11), 164.71 (C-16), 155.12 (C-5), 153.52 (C-18), 149.14 (C-4), 138.93 (C-7), 134.56 (C-10), 132.84 (C-20), 129.69 (C-21), 128.79 (C-27), 127.67 (C-24), 126.29 (C-26), 123.43 (C-25), 117.81 (C-19), 109.44 (C-23), 103.45 (C-2), 102.20 (C-13), 94.60 (C-6), 53.92 (C-14), 13.74 (C-15).

The corresponding naphthyl ester (**8**); 1-ethyl-6-fluoro-1,4-dihydro-4-oxo-7-(1-piperazinyl)-3-quinolinenaphthyl ester (C_26_H_24_FN_3_O_3_, mol wt 445.48), yield: 0.430 g of white needles (32%), m.p. 236–237 °C. I.R. (KBr): 3419, 1626, 1489, 1385, 1269, 1030, 931, 825, 741 cm^−1. 1^H-NMR (300 MHz, CDCl3): δ = ppm 8.65 (s, 1H, H-13), 8.07 (d, 1H, J_9–F_ = 9.20, H-9), 8.00 (m, 1H, H-23), 7.94 (m, 1H, H-22), 7.80 (m, 1H, H-30), 7.40-7.19 (m, 4H, H naphthyl-26, 27, 28, 29), 6.30 (d, 1H, J_16–F_ = 6.20, H-16), 4.31 (c, 2H, J_31–32_ = 6.88, H-31), 3.36–3.78 (m, 8H, H piperazin-6, 5, 3, 2), 2.29 (s, 1H, H-4), 1.57 (t, 3H, J_32–31_ = 6.88 H-32).

**Figure:**
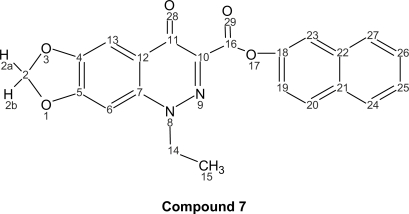


^13^C-NMR (300 MHz, CDCl_3_): δ = 176.94 (CO-11), 167.20 (CO-19), 159.40 (C-15), 155.20 (C-8), 149.20 (C-21), 147.77 (C-13), 146.38 (C-7), 137.09 (C-25), 137.00 (C-15), 131.00 (C-24), 127.50 (C-30), 127.52 (C-27), 125.50 (C-29), 125.00 (C-28), 120.50 (C-10), 120.48 (C-22), 118.00 (C-26), 112.94 (C-9), 108.37 (C-12), 105.02 (C-16), 99.75 (C-16), 51.06 (C-2, 6), 49.66 (C-31), 45.84 (C-3, 5), 14.39 (32).

The corresponding naphthyl ester (**9**); 1-cyclopropyl-6-fluoro-4-oxo-7-piperazin-1-yl-quinoline-3-naphthyl ester (C_27_H_24_FN_3_O_3_, mol wt 457.49), yield: 0.380 g of white needles (31%), m.p. 280–282 °C. I.R. (KBr): 3439, 1624, 1485, 1378, 1183, 1147, 1029, 944, 733, 621, 542 cm^−1^.

^1^H-NMR (300 MHz, CDCl_3_): δ = 8.77 (d, 1H, J_9–F_ = 8.70, H-9), 8.35 (d, 1H, J_23–22_ = 9.60, H-23), 8.20 (s, 1H, H-13), 8.00 (d, 1H, J_22–23_ = 9.60, H-22), 7.80 (m, 1H, H-30), 7.60 (d, 1H, J_26–22_ = 2.60, H-26), 7.50 (m, 1H, H-28), 7.40 (m, 1H, H-27), 7.20 (m, 1H, H-29), 6.30 (s, 1H, H-16), 3.30-2.70 (m, 8H, H piperazin-2, 6, 3, 5), 2.50 (s, 1H, H-4), 2.38 (dt, 1H, J_31–32_ = J_31–33_ = 5.00, H-31), 2.00 (m 2H, H-32), 1.90 (m, 2H, H-33).

^13^C-NMR (300 MHz, CDCl3): δ = 175.50 (CO-11), 160.10 (C-19), 156.10 (C-8), 150.00 (C-21), 143.00 (C-7), 142.20 (C-13), 138.05 (C-25), 137.00 (C-15), 132.8 (C-23), 131.42 (C-24), 127.60 (C-30), 127.47 (C-27), 126.48 (C-29), 125.50 (C-28), 120.00 (C-22), 116.62 (C-26), 116.45 (C-10), 109.80 (C-12), 107.25 (C-9), 106.50 (C-16), 51.30 (C-2, 6), 45.00 (C-3, 5), 28.45 (C-31), 7.50 (C-32, 33).

The corresponding naphthyl ester (**10**); 1-ethyl-6-fluoro-4-oxo-7-piperazin-1-yl-[1,8] naphthyridine-3-naphthyl ester (C_25_H_23_FN_4_O_3_, mol wt 446.47), yield: 0.461 g of white needles (34%), m.p. 239–240 °C. I.R. (KBr): 3439, 1624, 1485, 1378, 1293, 1183, 1147, 1029, 944, 733, 621, 542 cm^−1. 1^H-NMR (300 MHz, CDCl_3_): δ = ppm 8.35 (d, 1H, J_23-22_ = 9.00, H-23), 8.05 (s, 1H, H-11), 7.90 (d, 1H, J_22–23_ = 9.00, H-22), 7.80 (m, 1H, H-30), 7.61 (d, 1H, J_26–22_ = 2.50, H-26), 7.48 (m, 1H, H-28), 7.40 (d, 1H, J_15–F_ = 9.60, H-15), 7.38 (m, 1H, H-27), 7.12 (m, 1H, H-29), 4.60 (c, 2H, J_17–18_ = 6.80, H-17), 3.00-2.80 (m, 8H, H piperazin-6, 5, 2, 3), 2.20 (s, 1H, H-4), 1.38 (t, 3H, J_18–17_ = 6.80 H-18).

^13^C-NMR (300 MHz, CDCl_3_): δ = 176.00 (CO-13), 166.20 (CO-9), 160.00 (CO-19), 150.00 (C-21), 148.70 (C-7), 147.00 (C-11), 146.50 (C-16),139.00(C-25),133.00(C-23),131.05(C-24), 127.50 (C-30), 127.00 (C-27), 126.40 (C-29), 125.60 (C-28), 120.00 (C-22), 119.70 (C-15), 118.02 (C-26), 112.20 (C-14), 107.60 (C-12), 48.00 (C-2, 6), 47.10 (C-17), 45.88 (C-3, 5), 14.90 (C-18).

**Figure:**
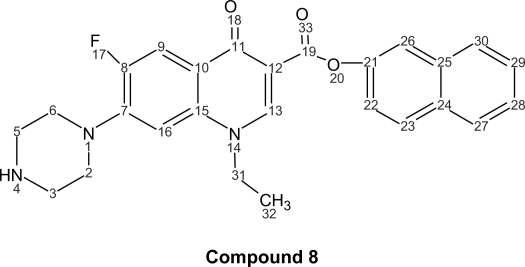


The irradiation conditions described in the experimental section were taken in order to closer resemble the conditions under which their photo-toxicity and the possible phototherapy applications are produced in biological media or *in vitro* or *in vivo* cellular systems.

The quantification of singlet oxygen (^1^O_2_) was carrying out using the methodology reported by Kraljic in 1978, for its detection (Kraljic and El Mohsni 1978). This methodology is based to the “bleaching” (as secondary reaction) of p-nitrosodimethylaniline (RNO) induced by the selective reaction of singlet oxygen with imidazol derived, in our case we used the histidine like this derived. In the reaction of ^1^O_2_ with histidine a *trans*-annular peroxide takes place as intermediary product, causing the “bleaching” of the group RNO, which can be followed to 440 nm. In absence of RNO the peroxide suffers a rearranges to produce the final products of oxygenation. This methodology can be applied for the quantification of singlet oxygen generated by photosensitizers, since the disappearance of the band of the RNO (to 440 nm) it is a direct measure of the quantity of ^1^O_2_ generated by them.

**Figure:**
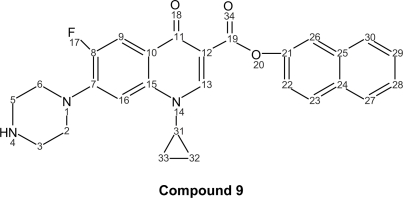


**Figure:**
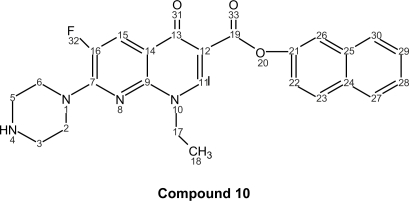


**Figure 2:**
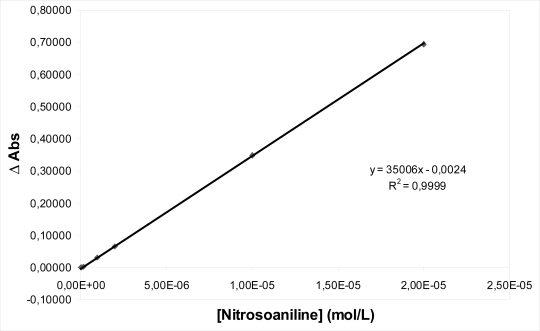
Calibration plot. Date are expressed as means ±S.E.M. Statistically significant (p < 0.05).

For the quantification based on this methodology we elaborated a calibration curve of the change of optic density as function of the change of p-nitrosodimethylaniline concentration. This curve presents a linear development until to a concentration of 2.00 × 10^−5^ mol/L (p-nitrosodimethylaniline). For each irradiation time, the variations of optic density were obtained, and by means of the calibration curve of the [p-nitrosodimethylaniline] variations (Fig. 2), they could be related these directly with the quantity of ^1^O_2_ taken place by each one of the studied species.

The statistical validation of this linear regression for the validation of the typical errors was performed at the 95% confidential level. The Table 1 shown the figures of merit obtained for the developed of the methodology. The detection limit (DL, 3*σ*) was calculated for the different irradiation times (45, 60 and 75 min).

## Quantification of ^1^O_2_ for the photosensitizers

Kraljic and collaborators report that the generation of ^1^O_2_ by photosensitizers is dependent of the irradiation time, as well as of the wave length used in the irradiation. In our case, the production of ^1^O_2_ by the compounds **6** to **10** were carried out using the same instrumentation and conditions described in the experimental section with irradiation times of 45, 60 and 75 min. The concentration used for each compound studied, for the generation of ^1^O_2_, was of 0,1 mM (H_2_O/ethanol, 90:10).

To verify the developed methodology, rose Bengal was used as standard. In the Table 2 the results are shown obtained for each one of the new compounds.

The results obtained for the quinolones **1** to **5** were not reported, because the production of ^1^O_2_ was below to the limit of detection of the developed methodology. It is advisable to be carried out by means of the direct measures of detection of the singlet oxygen emission by means of technical of flash photolysis with photomultiplier of germanium for example, although it turns out to be very expensive (Sortino and Scaiano 1999). The formation of singlet oxygen by the photosensitizing mechanism during the photolysis of photosensitizing compounds could be also evidenced by trapping with 2,5-dimethylfuran (GC-mass) (Gollnick and Griesbeck 1983; Costanzo et al 1989); 1,3-cyclohexadiene-1,4-diethanoate (HPLC) (Nardello et al 1997) as ^1^O_2_ scavengers, furfuryl alcohol (Haag et al 1984; Vargas et al 2001) but these no direct analytical methodology are imprecise and with methodology more complicated that the one developed in this paper on the detection base on histidine. In such a sense we can deduce that most of the phototoxicity taken place by the quinolones **1** to **5** could be produced for the most part by the formation of free radicals, superoxide anion and in smaller grade for singlet oxygen.

The ester compounds **6** to **10** are capable of producing singlet oxygen when it is irradiated with UV-A and visible light in the presence of molecular oxygen. This fact can be confirmed by trapping with histidine. We use a simple and sensitive spectrophotometric method for the detection of ^1^O_2_ as produced by different sensitizing dyes in neutral air saturated aqueous solutions. The reaction between histidine and ^1^O_2_ results in the formation of a trans-annular peroxide. The presence of the latter compound may be detected by bleaching the p-nitrosodimethylaniline at 440 nm. Singlet oxygen alone can not cause the bleaching of the latter compound. No bleaching occurs in the mixture of histidine and p-nitrosodimethylaniline without singlet oxygen (Kraljic and El Mohsni 1978). In order to control the reaction, we observe no measurable loss of the p-nitrosodimethylaniline in the absence of histidine.

With the obtained results we can also estimate relative to the φ(^1^O_2_) of the Rose Bengal = 0.76, the quantum yields of singlet oxygen to the naphthyl ester derivates. Being for the compound **6** = 0.034; to **7** = 0.052; **8** = 0.025; **9** = 0.023 and to compound **10** = 0.050.

We conclude that an oxidation of histidine (which is susceptible to singlet oxygen attack) is produced through photoexcitation of the ester derived compounds acting as a singlet oxygen sensitizer (type II mechanism). This particular reaction with histidine can be regarded as a model for damage to cellular protein inflicted by photoexcited quinolone antibacterials via formation of singlet oxygen.

On the basis of the results in the present investigation it can be concluded that an analytic method of histidine assay can be used safely to determine the generation of singlet oxygen by drugs.

We have proven that the naphthyl ester derivates of quinolones **1** to **5** produces singlet oxygen under irradiation with visible light. The compounds **7** > **10** > **6** > **8** > **9** (of major to minor generation of singlet oxygen) have the advantages such as sufficient strength to generate under irradiation with visible light ^1^O_2_, good hydrophilicity and therefore potential specific affinity for malignant tumors. These facts are of major significance for the study of its photodynamic action and make these compounds a promising candidate as PDT agents in the medical field. *In situ* production of the singlet oxygen could be the principle mechanism for tumor destruction in application of photodynamic therapy employing these novel water soluble compounds. Their photobiological properties are deserving of our further investigation.

We don’t dare to make any conclusion on a relationship between the structure of the compounds and the generation of singlet oxygen until having a studies it has more than enough theoretical parameters, for example those carried out on phototoxicity of antihyperlipoproteinemic drugs (Aguiar et al 1995).

## Figures and Tables

**Table 1:** Figures of merit.

**Regresion line:** Δ**O.D^a^** = **a**Δ**C^b^** + **b**			**D.L. (3***σ***) (**μ**g L**^−^**^1^)**		
a ± sa	b ± sb	r^2^	ct = 45	t = 60	t = 75
(350 ± 1) × 10^2^	−0,0024 ± 0.0009	0.99993	4,24	1,48	1,29

^a^ΔO.D: Variation of the optic density.

^b^ΔC: Variation of the p-nitrosodimethylaniline concentration.

^c^time of irradiation (t) in minutes.

**Table 2:** Generation of ^1^O2 by the compounds **6**–**10**.

**Compounds**	^1^**O**_2_ ± **SD (**μ**g L**^−^**^1^)**		
	**t = 45**	**t = 60**	**t = 75**
Rose Bengal	832 ± 1	886,9 ± 0,9	929 ± 2
**6**	38 ± 4	50 ± 5	70 ± 5
**7**	56,9 ± 0,1	76,5 ± 0,5	93,9 ± 0,3
**8**	28,0 ± 0,7	40,0 ± 0,2	52,8 ± 0,9
**9**	24,9 ± 0,5	27,1 ± 0,7	37,7 ± 0,7
**10**	55,6 ± 0,3	70,9 ± 0,1	84 ± 1
